# Supplementary material for: Causal modelling of heavy-tailed variables and confounders with application to river flow
Source: Extremes (Boston). 2022 Dec 17;26(3):573–94. doi: 10.1007/s10687-022-00456-4 (PMC10423152; doi:10.1007/s10687-022-00456-4)
Supplement: Supplementary file 1 — Supplementary file1 (PDF 196 KB) [file 10687_2022_456_MOESM1_ESM.pdf]

SUPPLEMENTARY MATERIAL TO

"CAUSAL MODELLING OF HEAVY-TAILED VARIABLES AND CONFOUNDERS WITH APPLICATION TO RIVER FLOW"

Olivier C. Pasche
Research Center for Statistics, University of Geneva, Switzerland,
Institute of Mathematics, EPFL, 1015 Lausanne, Switzerland,
`olivier.pasche@unige.ch`

Valérie Chavez-Demoulin
Faculty of Business and Economics, University of Lausanne, Switzerland,
`valerie.chavez@unil.ch`

Anthony C. Davison
Institute of Mathematics, EPFL, 1015 Lausanne, Switzerland,
`anthony.davison@epfl.ch`

S.1 Variables with Comparable Tails

S.1.1 Non-Parametric Causal Tail Coefficient Estimator

Figure S.1 shows the sample distributions of the non-parametric estimators $\hat{\Gamma}_{1,2}$ and $\hat{\Gamma}_{2,1}$ for all four causal structures, for the t_4, $\mathrm{Pareto}(1,2)$ and $\mathrm{LogN}(0,1)$ noise distributions, respectively. The true coefficient values $\Gamma_{1,2}$ and $\Gamma_{2,1}$ are obtained using (2). Figure S.2 shows the sample distribution of the coefficient difference estimator $\hat{\Delta}_{1,2} := \hat{\Gamma}_{1,2} - \hat{\Gamma}_{2,1}$ for the t_4 noise distribution.

S.1.2 LGPD Causal Tail Coefficient with Post-Fit and Constrained Fit Corrections

Figure S.3 shows the sample distribution of $\hat{\Gamma}^{\mathrm{GPD}}_{1,2|H}$ and $\hat{\Gamma}^{\mathrm{GPD}}_{2,1|H}$ with the constrained fit, for a comparable confounder tail. Figure S.4 shows the sample distribution of $\hat{\Gamma}^{\mathrm{GPD}}_{1,2|H}$ and $\hat{\Gamma}^{\mathrm{GPD}}_{2,1|H}$ with post-fit correction for all four causal configurations, for the t_4, $\mathrm{Pareto}(1,2)$ and $\mathrm{LogN}(0,1)$ noise distributions.

S.2 Application Results for Competitors

Table S.1 shows the causal coefficients between the discharge station pairs estimated using ICA-LiNGAM, with and without considering the average catchment precipitation variable.

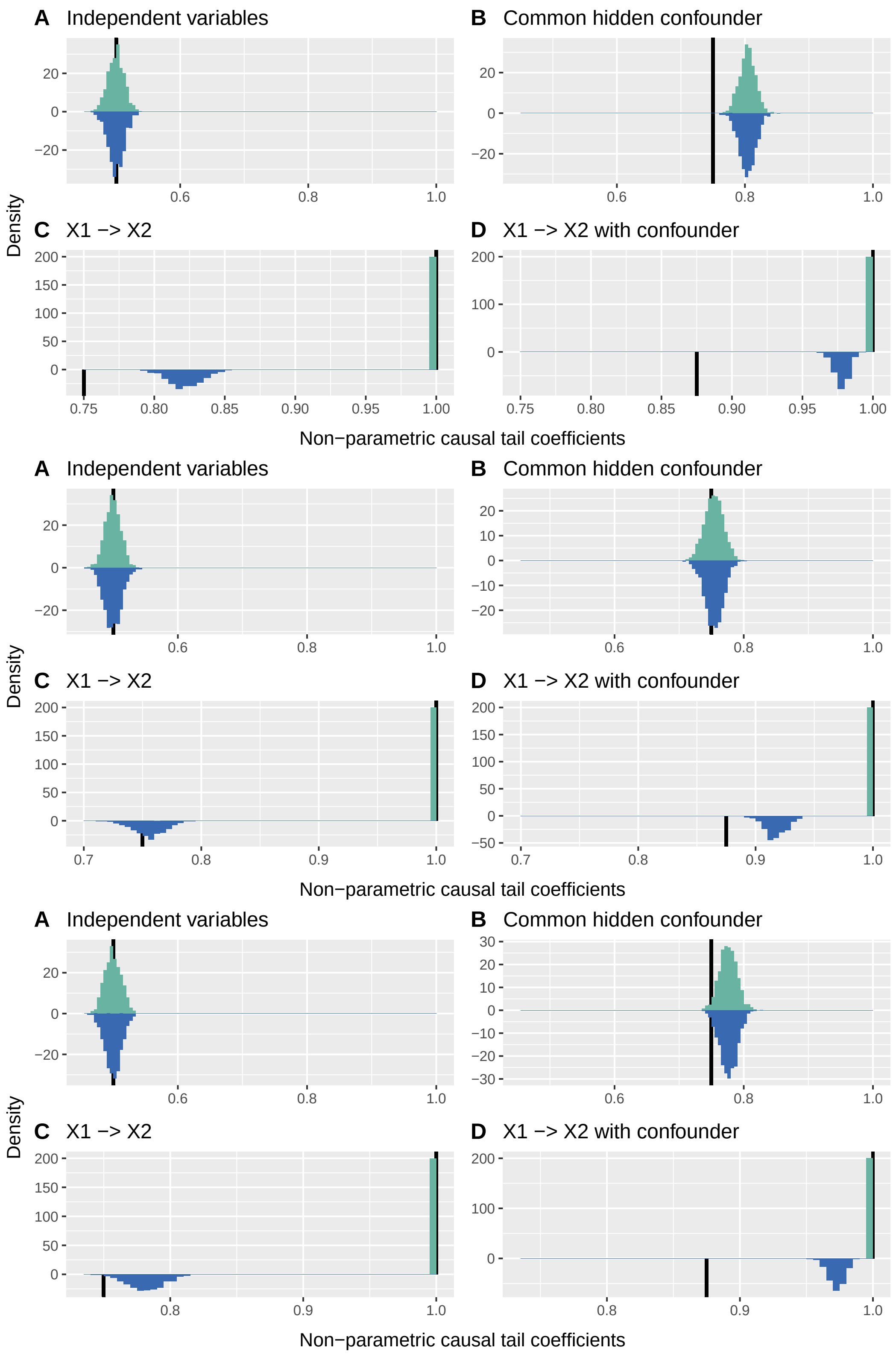

Figure S.1: Histograms of $\hat{\Gamma}_{1,2}$ (turquoise) and $\hat{\Gamma}_{2,1}$ (blue) for t_4 (top four panels), $\mathrm{Pareto}(1,2)$ (middle four panels) and $\mathrm{LogN}(0,1)$ (bottom four panels) distributed noise variables, for the four causal configurations. Half-lines (black) indicate $\Gamma_{1,2}$ and $\Gamma_{2,1}$.

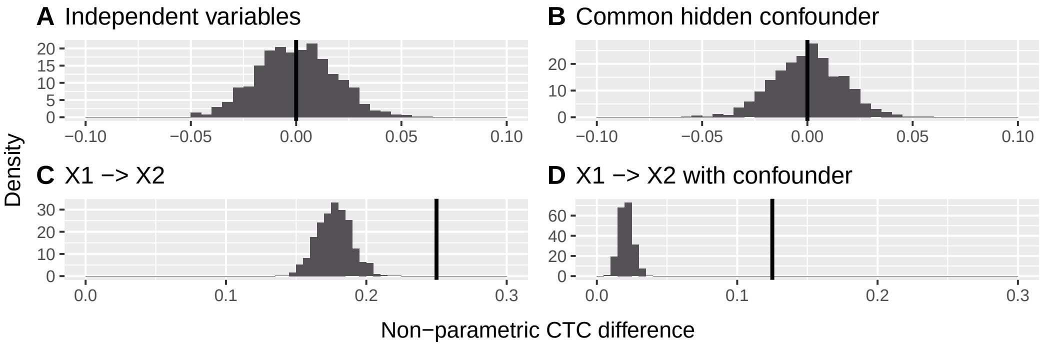

Figure S.2: Histogram of $\hat{\Delta}_{1,2}$ for t_4 distributed noise variables, for the four causal configurations. Lines indicate $\Delta_{1,2} = \Gamma_{1,2} - \Gamma_{2,1}$.

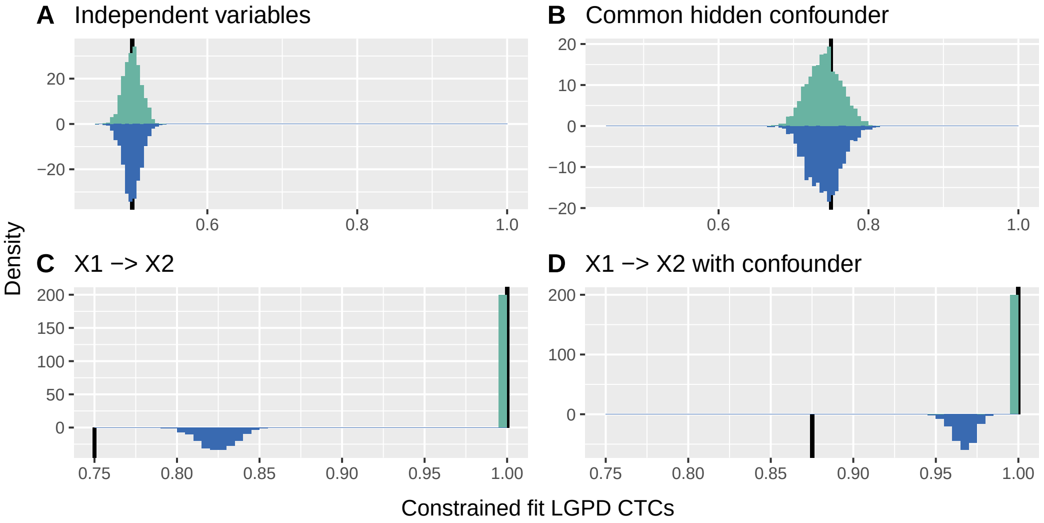

Figure S.3: Histograms of $\hat{\Gamma}^{\mathrm{GPD}}_{1,2|H}$ (turquoise) and $\hat{\Gamma}^{\mathrm{GPD}}_{2,1|H}$ (blue) with constrained fit for t_4 distributed noise variables, for the four causal configurations. Half-lines (black) indicate $\Gamma_{1,2}$ and $\Gamma_{2,1}$.

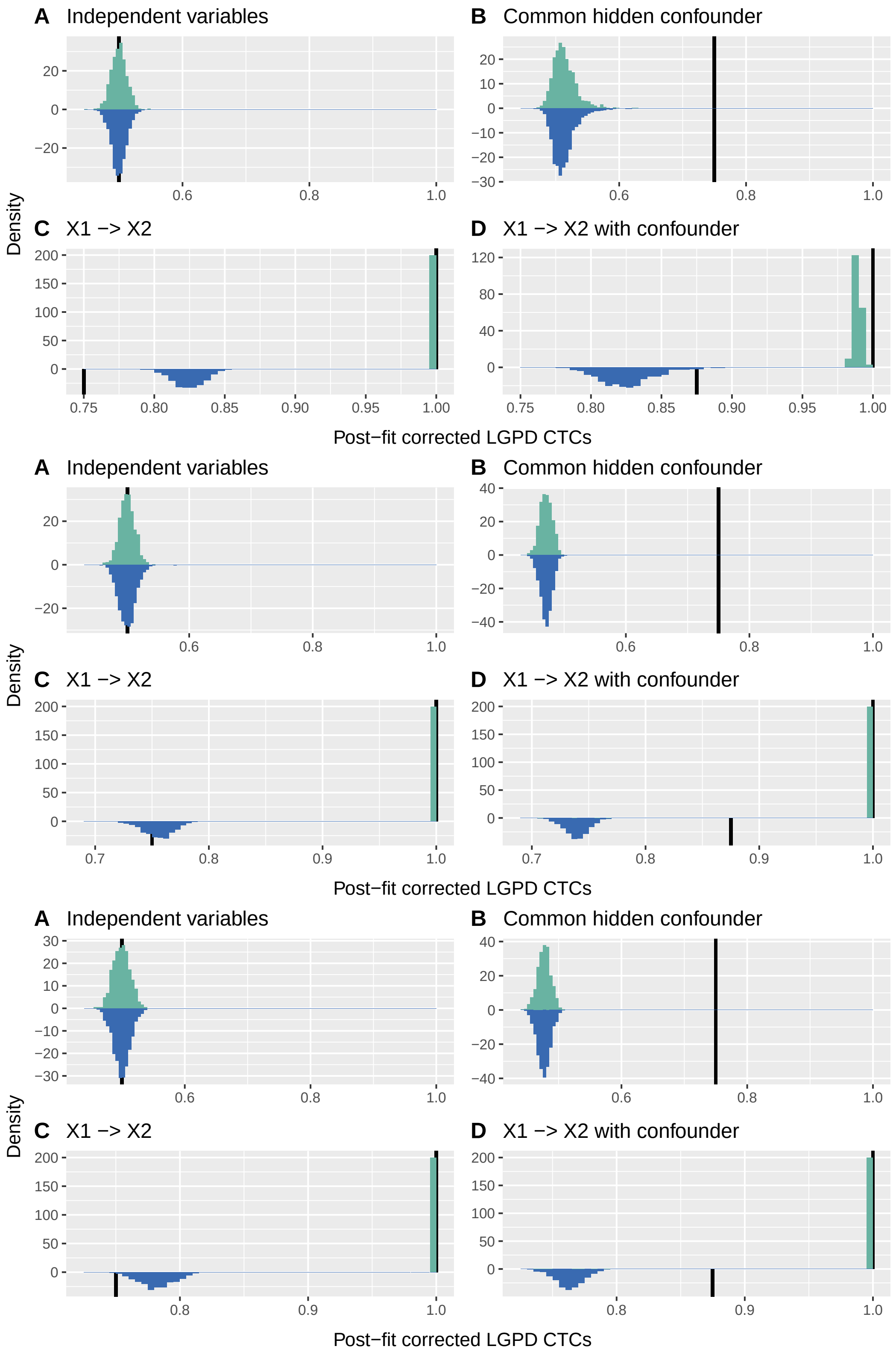

Figure S.4: Histograms of $\hat{\Gamma}^{\mathrm{GPD}}_{1,2|H}$ (turquoise) and $\hat{\Gamma}^{\mathrm{GPD}}_{2,1|H}$ (blue) with post-fit correction for t_4 (top four panels), $\mathrm{Pareto}(1, 2)$ (middle four panels) and $\mathrm{LogN}(0, 1)$ (bottom four panels) distributed noise variables, for the four causal configurations. Half-lines (black) indicate $\Gamma_{1,2}$ and $\Gamma_{2,1}$.

Table S.1: Linear causal coefficients for the discharge station pairs estimated with the ICA-LiNGAM algorithm using either the station pair only (LiNGAM, two variables) or the station pair and precipitation (LiNGAM-H, three variables). Non-null values indicate significant causal effects. The arrows indicate the estimated direct causal directions between the stations.

Stations	Pair type	LiNGAM	LiNGAM-H
43-62	causal	$1.92^{\rightarrow}$	$2.02^{\rightarrow}$
42-63	causal	$2.08^{\rightarrow}$	$2.21^{\rightarrow}$
36-63	causal	$3.29^{\rightarrow}$	$3.61^{\rightarrow}$
24-61	causal	$2.96^{\rightarrow}$	$3.03^{\rightarrow}$
44-61	causal	$2.66^{\rightarrow}$	$2.83^{\rightarrow}$
22-38	causal	$2.35^{\rightarrow}$	$2.35^{\rightarrow}$
22-35	causal	$2.55^{\rightarrow}$	$2.55^{\rightarrow}$
30-45	non-caus.	$0.84^{\rightarrow}$	$0.87^{\rightarrow}$
36-39	non-caus.	$0.66^{\leftarrow}$	$0.66^{\leftarrow}$
42-34	non-caus.	$1.39^{\leftarrow}$	$1.29^{\leftarrow}$
42-34*	non-caus.	$1.39^{\leftarrow}$	$1.39^{\leftarrow}$
32-33	non-caus.	$0.59^{\rightarrow}$	$0.54^{\rightarrow}$
62-63	non-caus.	$1.02^{\rightarrow}$	$1.05^{\rightarrow}$
57-60	non-caus.	$0.68^{\rightarrow}$	$0.67^{\rightarrow}$
13-14	non-caus.	$0.50^{\leftarrow}$	$1.10^{\rightarrow}$
17-22	non-caus.	$1.80^{\rightarrow}$	$1.69^{\rightarrow}$
12-21	non-caus.	$1.04^{\rightarrow}$	$1.08^{\rightarrow}$
26-28	non-caus.	$0.75^{\leftarrow}$	$0.72^{\leftarrow}$
27-31	non-caus.	$0.54^{\rightarrow}$	$0.66^{\rightarrow}$
23-39	non-caus.	$0.25^{\rightarrow}$	$0.18^{\rightarrow}$
23-35	non-caus.	$0.42^{\rightarrow}$	$0.36^{\rightarrow}$
